# Chronic Ultrasound Prenatal Stress Altered the Brain's Neurochemical Systems in Newborn Rats

**DOI:** 10.1155/2024/3829941

**Published:** 2024-02-13

**Authors:** Olga Abramova, Yana Zorkina, Konstantin Pavlov, Valeria Ushakova, Anna Morozova, Eugene Zubkov, Olga Pavlova, Zinaida Storozheva, Olga Gurina, Vladimir Chekhonin

**Affiliations:** ^1^Department of Basic and Applied Neurobiology, V. Serbsky National Medical Research Centre of Psychiatry and Narcology, Moscow, Russia; ^2^Department of Biology, Lomonosov Moscow State University, Moscow, Russia; ^3^Mental-Health Clinic No. 1 Named After N.A. Alekseev, Zagorodnoe Highway 2, Moscow 115191, Russia; ^4^Laboratory of Functional Neurochemistry, P. K. Anokhin Institute of Normal Physiology, Moscow, Russia; ^5^Department of Medical Nanobiotechnology, Pirogov Russian National Research Medical University, Moscow, Russia

## Abstract

Prenatal stress (PS) affects the development and functioning of the central nervous system, but the exact mechanisms underpinning this effect have not been pinpointed yet. A promising model of PS is one based on chronic exposure of pregnant rodents to variable-frequency ultrasound (US PS), as it mimics the PS with a psychic nature that most adequately captures the human stressors in modern society. The aim of this study was to investigate the effects of US PS on the brain neurotransmitter, neuropeptide, and neurotrophic systems of newborn Wistar rats. We determined the concentration of neurotransmitters and their metabolites (serotonin, HIAA, dopamine, DOPAC, and norepinephrine), neuropeptides (*α*-MSH, *β*-endorphin, neurotensin, oxytocin, and substance P), and the neurotrophin brain-derived neurotrophic factor (BDNF) in rat brain tissues by HPLC-ED, ELISA, and multiplex ELISA. Correlation analysis and principal component analysis (PCA) were used to get a sense of the relationship between the biochemical parameters of the brain. The results demonstrated that US PS increases the concentration of serotonin (*p*=0.004) and DOPAC (*p*=0.04) in the hippocampus has no effect on the neurotransmitter systems of the frontal cortex, reduces the concentration of BDNF in the entirety of the brain of males (*p*=0.008), and increases the neuropeptides *α*-MSH (*p*=0.02), *β*-endorphin (*p*=0.01), oxytocin (*p*=0.008), and substance P (*p* < 0.001) in the entire brain. A degree of complexity in the neurotransmitter system network in the frontal cortex and network change in the hippocampus after exposure to US PS have been observed. PCA revealed a similar pattern of neurotransmitter system interactions in the frontal cortex and hippocampus in males and females after exposure to US PS. We suggest that US PS can alter neurodevelopment, which is mediated by changes in the studied neurochemical systems that thus affect the behavioral phenotype in animals.

## 1. Introduction

The embryonic brain develops and matures rapidly during the prenatal period [1, 2]. The biological signals the brain receives can affect its development. This includes endogenous biological signals from the mother that comprise signals mediated by maternal stress. Such exposure leads to prenatal stress (PS) in the offspring [1–3]. Changes in fetal nervous system development under the influence of PS may persist after birth and manifest themselves at the behavioral, physiological, and molecular levels; the offspring can suffer structural and functional impairments to their brain after PS, which eventually can lead to persistent behavioral phenotype variations [1, 3, 4].

The exact mechanisms by which PS affects offspring remain unknown [5]. One of the possible ways through which PS affects brain development and function is by impairing the functioning of the brain's neurotransmitter systems. Monoamines (serotonin, dopamine, and norepinephrine) are the most functionally important neurotransmitters that are well-characterized [6]. The serotonergic system is formed during early fetal development and innervates almost the entire central nervous system (CNS) [7]. Serotonin regulates the development of the nervous system by modulating neurogenesis and cell proliferation and influencing brain wiring during prenatal and early postnatal development, which ensures proper brain function [8, 9]. Stressful events and anxiety disorders during pregnancy alter serotonin levels in the fetal brain, potentially leading to disruption in the formation of interneuronal connections [4, 10, 11]. Norepinephrine is another neurotransmitter that is important for normal brain development, including the cortex. Disruption of the noradrenergic system during the prenatal period can lead to impaired astrogliosis and glial proliferation, as well as to alterations in the dendrites [12]. The dopaminergic system is also associated with brain ontogenesis. It is believed that dopamine has an important stabilizing and integrative influence on interneuron communication. Disruption of the dopaminergic system by various factors can destabilize neuron connections in the brain [13]. Another important factor that may be involved in PS mechanisms is the brain-derived neurotrophic factor (BDNF). BDNF is involved mainly in neurogenesis, gliogenesis, synaptogenesis, and the regulation of neuronal connections during brain development [14–16]. PS possibly impairs these important processes through a general decrease in the level of BDNF biosynthesis [17]. Studies have demonstrated that PS suppresses cell proliferation and affects the processes of neurogenesis, neuronal migration, and cell differentiation in the fetal brain. This leads to long-term persistent effects on various areas of the brain. It is believed that these changes are associated with BDNF [15, 18, 19]. Neuropeptides are other potentially important but less studied elements in the mechanism of PS action on the CNS. Neuropeptides represent auxiliary signaling molecules in the CNS that coexist with classical neurotransmitters [20]. The neuropeptide class of molecules is more diverse than other signaling molecules. It is assumed that neuropeptides mediate a more prolonged modulation of neural processes [21]. The combined effects of different neuropeptide systems play a central role in the complex regulation of behavior [22].

Therefore, various biochemical mechanisms could potentially be associated with the mechanism of PS action on brain development, which has yet to be fully understood. However, the instruments and methods used to study the action of PS are also important. PS models on laboratory animals are widely used in biomedical experimental studies, since human studies are limited. Such models can sufficiently replicate the features of PS action at the physiological and molecular levels. However, the results of fetal programing depend on many factors, for example, time and duration of the exposure, type, and intensity of the stimulus, etc., [4]. Stress factors can be of varying nature, but pregnant women in modern society are more often exposed to psychological stress (worry about their children, financial stress, work issues, etc.) than to physical stress. This should be taken into account during the study of PS on animal models and the selection of the most appropriate ways of stressing [23]. Different ways of stressing pregnant females are used to replicate PS in animals, of which the most common are restraint stress, the administration of glucocorticoids or chronic unpredictable mild stress [24]. However, these methods do not emphasize the main stress factor in modern society—information stress. Exposure of animals to variable frequency ultrasound (US) is promising in replicating the effects of informational stress on rodents. The effectiveness of US use for stressing rodents has been demonstrated in several studies [25, 26]. In addition, it has been previously demonstrated that unavoidable exposure of pregnant female rats to US during the entire gestation causes alteration of the behavioral phenotype of the offspring toward psychopathology [27–29]. The US effect is based on the fact that rodents communicate using US and that signals of different frequencies contain certain information for them. For example, baby rats emit 40 kHz US signals when isolated from their mothers, and adults emit 22–25 kHz signals in threatening situations. Takahashi et al. [30]. The US stress model consists of alternate exposure of rodents to these frequency signals, which have a negative informational content. Additionally, mention should be made of medical technologies using US that may affect the pregnant woman. Ultrasonography is known to be widely used for prenatal screening during pregnancy, and its safety for the fetus has been previously shown [31, 32]. A question may arise about the identity of ultrasonography US exposure and our model of US stress, but here, it is necessary to point out the different principles of these effects. US signals have an informational nature for rodents and thus can become, for them, a stressor of a psychic nature, unlike humans, which do not distinguish these signals. In addition, it should be kept in mind that US information signals can only directly affect the pregnant female and not the fetus, since in rats, the onset of auditory function, monitored by recording responses from the auditory nerve and brainstem, begins on postnatal day (PND) 12–15 and reaches thresholds at about PND 22 [33–35]. In addition, newborn rats have an air-bone gap between the outer and middle ear that closes by approximately 15 PND [34]. Therefore, newborn rats exhibit both sensory-neural and conductive immaturity of hearing until at least 15 PND [34], which prevents them from picking up sound signals until this age. Therefore, US PS acts on the fetus indirectly, through maternal psychic stress during pregnancy. However, the biochemical and molecular mechanisms of this phenomenon have not yet been studied.

The purpose of this study was to investigate the neurotransmitter, neuropeptide, and neurotrophic brain systems of newborn rats that had been exposed to PS induced by exposure of pregnant female rats to variable frequency US (US PS). Since US PS leads to alterations in the behavioral phenotype of rats, we hypothesized that that may be due to the modifications of these systems during prenatal development. For this purpose, in our study, we analyzed the concentrations of a series of biochemical parameters in newborn rats at the age of 1 day. Additionally, the results of our study have some practical value because we have previously suggested that it is possible to create a model of psychopathology based on prenatal exposure to US PS [27, 29]. The obtained results provided important clues for determining the features of the assumed model, along with previous studies.

## 2. Materials and Methods

### 2.1. Experimental Animals

The experiment involved Wistar rats obtained from the Laboratory Animal Nursery (Pushchino, Moscow region). We used 12 females from which we received offspring (*n* = 112). The rats were preliminarily adapted for living in the laboratory vivarium after moving from the nursery for 7 days before the experiments. Housing conditions and all experimental procedures were in accordance with the local ethical committee of the V.P. Serbsky National Medical Research Center for Psychiatry and Narcology and Directive 2010/63/EC of September 22, 2010. During the experiment, all animals stayed in the laboratory vivarium under constant conditions: lighting regime—12/12 hr, ambient room temperature –22°C, and free access to drinking water and food.

### 2.2. Experiment Design

Rat offspring for the experiment were obtained as follows: adult females (age 3 months; weight 180–200 g) were mated with males. Pairs were randomized so that there was one male for every two females. After mating with males, we obtained daily vaginal swabs from females to assess the presence of spermatozoa in it, which was considered the moment of fertilization and day zero of pregnancy. We assigned two females from each male to two groups randomly: control and stress. After fertilization, we housed all females in individual cages (53 cm × 35 cm × 19 cm). We placed females from the stress group under experimental US exposure for the entire gestation period immediately after fertilization. These females subsequently produced offspring with PS effects (PS offspring). Control females were housed in individual cages under standard conditions without experimental effects during the whole pregnancy. We obtained control offspring from this group of females. We housed all born females in individual cages with their pups under standard conditions and without experimental effects. Therefore, we obtained four groups of offspring: control males (*n* = 34), control females (*n* = 31), PS males (*n* = 22), and PS females (*n* = 25).

The pups were selected randomly from each experimental group and sacrificed 1 day after birth to collect brain tissue samples for biochemical analysis. For sampling, pups from each female were divided into two subgroups roughly equal by number of animals and sex. The frontal cortex and hippocampus from both cerebral hemispheres were sampled from the first subgroup (10 control males, 10 control females, 10 PS males, 10 PS females). A total of 40 hippocampal and 40 frontal cortex samples from the first subgroup were obtained for the experiment. The whole brain was taken from the second subgroup (10 control males, 10 control females, 10 PS males, 10 PS females). A total of 40 whole brain samples from the second subgroup were obtained for the experiment. The quantitative content of the metabolic parameters of neurotransmitter systems (serotonin, HIAA, dopamine, DOPAC, and norepinephrine) was determined in the frontal cortex and hippocampus. The quantitative content of BDNF and neuropeptides (*α*-melanocyte-stimulating hormone (*α*-MSH), *β*-endorphin, neurotensin, oxytocin, and Substance P) was evaluated in the whole brain.

### 2.3. Experimental Exposure to Variable Frequency US

We exposed pregnant rats to US continuously throughout the entire gestation period. We used a US generator to produce the US signals. The US frequency range alternated between the following frequencies: high frequencies (40–45 kHz), medium frequencies (25–40 kHz) and short frequencies (20–25 kHz). The change of frequencies occurred every 10 min. The total duration of exposure to high frequencies was 30%, short and medium frequencies were 35% each. The sound pressure level was maintained at 50 ± 5 dB (fluctuation ± 10%). We placed the US generator above the cages with animals at a height of 1 m from the cages. We changed the location of cages under the US generator daily [25, 26, 28, 36].

### 2.4. Collection and Storage of Biomaterials

Rat brain samples for the analysis of biochemical parameters concentrations were collected after brief anesthesia, followed by rapid decapitation and brain extraction between 12:00 and 14:00. Sterile Eppendorf was used to store the samples. Depending on the animal subgroup, the frontal cortex and hippocampus from both hemispheres or the whole brain were isolated after the brain extraction. Brain samples were placed in the Eppendorf, frozen in liquid nitrogen immediately after the isolation, and stored in liquid nitrogen until the analysis [37].

### 2.5. Evaluation of Neurotransmitter Concentrations

In scientific research, various methods are used to determine the concentration of neurotransmitters, for example, liquid chromatography, biosensors, electrochemical methods using micro- and nanoelectrodes, and mass spectrometry [38–40]. In our study, we assessed the concentrations of 5-hydroxyindoleacetic acid (HIAA), serotonin, norepinephrine, dopamine, 3,4-dihydroxyphenylacetic acid (DOPAC) (below—analytes) in the frontal cortex and hippocampus of rats by HPLC-ED. The samples were homogenized with 50 pmol/mL internal standard 3, 4-dihydroxybenzylamine hydrobromide (Sigma Aldrich, USA) using the ultrasonic homogenizer (Sartorius, France) in 0.1 n HClO_4_ (Sigma Aldrich, USA). We centrifuged the resulting solution for 15 min at 15,000 rpm. We performed HPLC separation using the reversed-phase column ReproSil-Pur, ODS-3, 4 × 100 mm with a pore diameter of 3 *µ*m (Dr. Majsch, Germany) at +28°C and a mobile phase speed of 1 mL/min supported by the liquid chromatograph LC-20ADsp (Shimadzu, Japan). The mobile phase consisted of 0.1 M citrate-phosphate buffer, 0.3 mM sodium octane sulfonate, 0.1 mM EDTA, and 8% acetonitrile (all reagents from Sigma Aldrich, USA), pH 2.58. The electrochemical detector Decade II (Antec Leyden, The Netherlands) was equipped with a working glassy carbon electrode (+0.80 V) and an Ag/AgCl reference electrode. We identified the peaks of interest and the internal standard by their release time in the standard solution. We used the internal standard method using a calibration curve with LabSolutions software (Shimadzu, Japan) to calculate the concentration of analytes. Brain tissue weight data were used to normalize samples [29, 41]. We determined DOPAC/dopamine and HIAA/serotonin ratios from concentration data as indicators of the metabolic turnover of dopamine and serotonin, respectively [42].

### 2.6. Sample Preparation of the Whole Brain Homogenate

To evaluate BDNF and neuropeptide concentrations, the whole rat brain homogenate was prepared. The homogenate was prepared as follows: the frozen tissue sample was weighed and dissolved in 5 mL of special buffer (Tris buffer, NaCl, EDTA, Tween 20, PMSF Protease Inhibitor (Thermo Scientific™, USA)) using the SilentCrusher S homogenizer (Heidolph, Germany), then centrifuged for 20 min at 16,000 rpm; the supernatant was collected, aliquoted, frozen, and stored at −80°C until the analysis [37, 43].

### 2.7. Immunoassay of BDNF Concentration

For the measurement of BDNF concentrations, brain samples homogenate was previously unfrozen at room temperature, and the BDNF level was determined using the commercial Rat BDNF ELISA Kit Cat. No: 3030003 (BioAim Scientific Inc), according to the manufacturer's protocol [44]. BDNF concentration was calculated per mg of tissue weight.

### 2.8. Multiplex Immunoassay of Neuropeptide Concentrations

The concentrations of *α*-MSH, *β*-endorphin, neurotensin, oxytocin, and substance P were determined in previously unfrozen whole brain homogenate using the commercial MILLIPLEX® Rat/Mouse Neuropeptide Magnetic Bead Panel (Cat. No. RMNPMAG-83K). The prepared homogenate was further processed by acetonitrile precipitation before the analysis. 600 *µ*L of acetonitrile was added to 400 *µ*L of brain homogenate and centrifuged at 17,000 × *g* for 5 min. Then, 500 *µ*L of the supernatant was taken, and the samples were dried at maximum vacuum (Concentrator plus, Eppendorf, Germany). The samples were reconstituted with 60 *µ*L Assay Buffer from the kit, then shaken for 10 min. Further analysis of the concentrations was performed according to the manufacturer's protocol. The measurements were performed on the Luminex® 200 with xPONENT® software. The median fluorescence intensity was analyzed using a weighted 5-parametric logistic method to calculate the analyte concentrations in the samples. The neuropeptide concentration was calculated per mg of tissue weight [37, 43].

### 2.9. Statistics

We performed statistical analysis of the obtained data and also made plotting with the freeware software—RStudio (Version 1.4, RStudio PBC, USA) and jamovi (Version 1.6, The jamovi project, Australia). We assessed the normality of the distribution of the obtained data using the Shapiro–Wilk test and determined that several indicators had a normal data distribution: HIAA concentration in the frontal cortex; also, serotonin concentration, HIAA concentration, and HIAA/serotonin in the hippocampus. The following indicators had an abnormal distribution of data: serotonin concentration, HIAA/serotonin ratio, dopamine concentration, DOPAC concentration, DOPAC/dopamine ratio, and norepinephrine concentration in the frontal cortex; also, dopamine concentration, DOPAC concentration, DOPAC/dopamine ratio, and norepinephrine concentration in the hippocampus; also, BDNF concentration, *α*-MSH concentration, *β*-endorphin concentration, neurotensin concentration, oxytocin concentration, and substance P concentration in the whole brain. The *p*-values were calculated using the two-way analysis of variance (ANOVA), followed by Tukey's test for multiple comparisons in the case of a normal distribution of data. We used the nonparametric Kruskal–Wallis (KW) test and the Mann–Whitney *U* test with Dwass–Steel–Critchlow–Fligner for multiple comparisons in case of nonnormally distributed data. We presented the results in the figures as mean ± SEM or Med (Q1; Q3) in the case of normal and nonnormal data distribution, respectively. A *p*-value < 0.05 was considered statistically significant.

The correlation analysis of the relationships between biochemical parameters was performed separately for two subgroups of offspring (whole brain and frontal cortex/hippocampus). We calculated Spearman's rank correlation coefficient to identify correlation relationships between different parameters. A *p* value < 0.01 was accepted as statistically significant to account for multiple comparisons in the correlation analysis [45].

Data were normalized and used for principal component analysis (PCA) in RStudio. Samples were compared by group and sex.

## 3. Results

### 3.1. Neurotransmitter Systems Analysis in the Frontal Cortex

**Figure 1:**
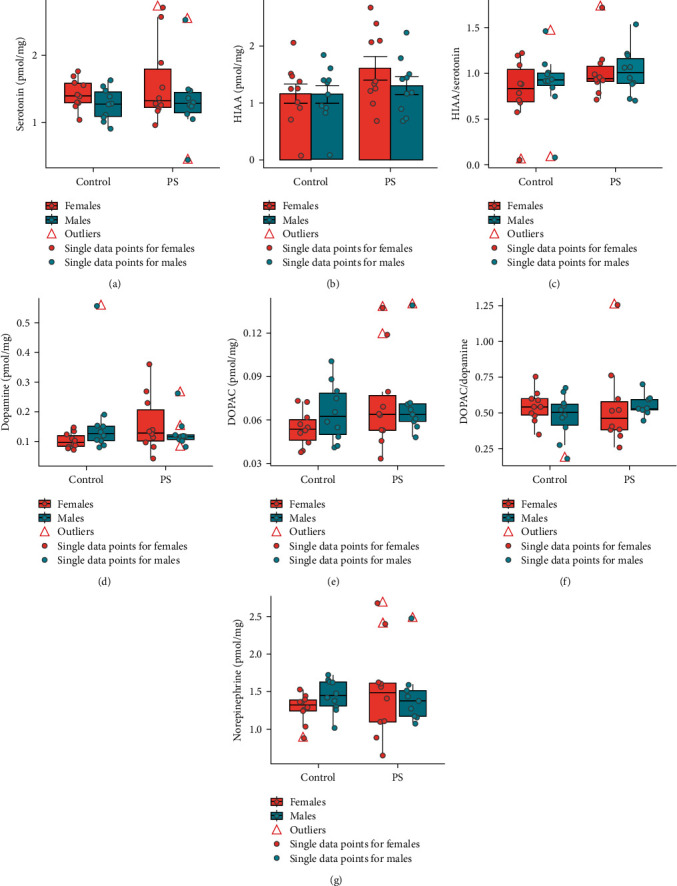
The metabolic parameters of neurotransmitter systems in the frontal cortex of newborn rats did not alter after exposure to ultrasound prenatal stress (*n* = 40 : 10 control males, 10 control females, 10 PS males, 10 PS females): (a) serotonin concentration in the frontal cortex; (b) HIAA concentration in the frontal cortex; (c) HIAA/serotonin ratio in the frontal cortex; (d) dopamine concentration in the frontal cortex; (e) DOPAC concentration in the frontal cortex; (f) DOPAC/dopamine ratio in the frontal cortex; (g) norepinephrine concentration in the frontal cortex. Data are expressed as med (Q1; Q3) in boxplots (a, c, d, e, f, and g) or as mean ± SEM in bar chat (b). Red triangles—outliers; colored dots—single data points (red—females, blue—males); red boxes—data on females; blue boxes—data on males; control, control group; PS, prenatal stress group; DOPAC, 3,4-dihydroxyphenylacetic acid; HIAA, 5-hydroxyindoleacetic acid.

Serotonin concentration (KW: *χ*^2^ = 2.55; *p*=0.47; Figure 1(a)), HIAA (ANOVA: *F* = 1.53; *p*=0.22; Figure 1(b)), HIAA/serotonin ratio (KW: *χ*^2^ = 2.78; *p*=0.43; Figure 1(c)), dopamine concentration (KW: *χ*^2^ = 3.89; *p*=0.27; Figure 1(d)), DOPAC (KW: *χ*^2^ = 3.07; *p*=0.38; Figure 1(e)), DOPAC/dopamine ratio (KW: *χ*^2^ = 1.98; *p*=0.57; Figure 1(f)), and norepinephrine concentration (KW: *χ*^2^ = 2.61; *p*=0.46; Figure 1(g)) in the frontal cortex of the offspring did not differ statistically between the groups.

### 3.2. Neurotransmitter Systems Analysis in the Hippocampus

**Figure 2:**
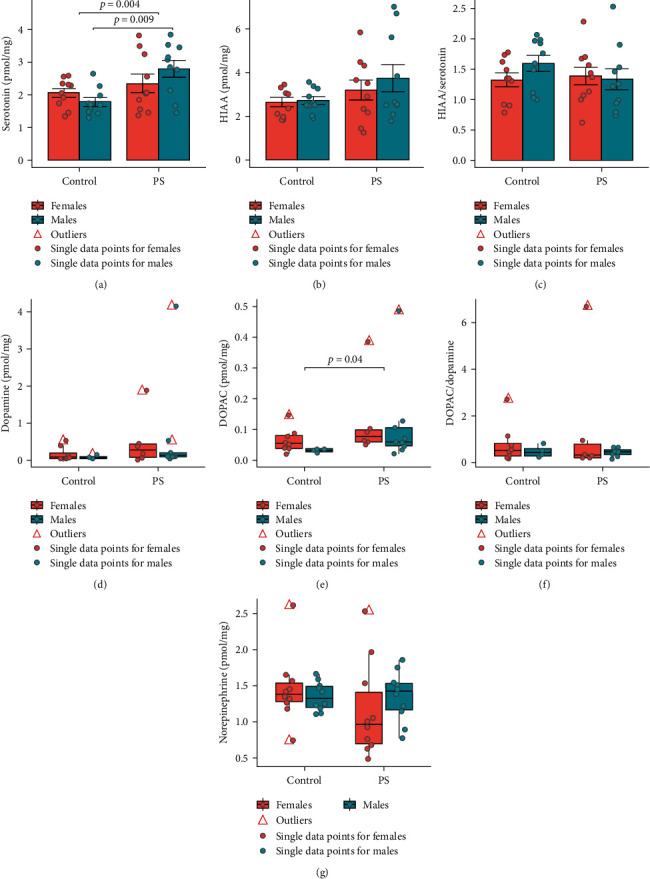
The metabolic parameters of neurotransmitter systems in the hippocampus of newborn rats underwent some changes after exposure to ultrasound prenatal stress (*n* = 40 : 10 control males, 10 control females, 10 PS males, 10 PS females): (a) serotonin concentration in the hippocampus; (b) HIAA concentration in the hippocampus; (c) HIAA/serotonin ratio in the hippocampus; (d) dopamine concentration in the hippocampus; (e) DOPAC concentration in the hippocampus; (f) DOPAC/dopamine ratio in the hippocampus; (g) norepinephrine concentration in the hippocampus. Data are expressed as med (Q1; Q3) in boxplots (d, e, f, and g) or as mean ± SEM in bar chat (a, b, and c). Red triangles—outliers; colored dots—single data points (red—females, blue—males); red boxes—data on females; blue boxes—data on males; control, control group; PS, prenatal stress group; DOPAC, 3,4-dihydroxyphenylacetic acid; HIAA, 5-hydroxyindoleacetic acid.

ANOVA analysis demonstrated significant differences between the groups by the hippocampal serotonin concentration (ANOVA: *F* = 4.18; *p*=0.01). PS offspring showed elevated serotonin concentrations compared to the control offspring (ANOVA: *F* = 9.45; *p*=0.004), but post hoc analysis revealed significant differences only for males (*p*=0.009): females (*p*=0.77) (Figure 2(a)). However, HIAA concentration (ANOVA: *F* = 1.50; *p*=0.23; Figure 2(b)) and HIAA/serotonin (ANOVA: *F* = 0.77; *p*=0.52; Figure 2(c)) in the hippocampus did not differ statistically between the groups.

Dopamine concentration (KW: *χ*^2^ = 2.77; *p*=0.43; Figure 2(d)) and DOPAC/dopamine ratio (KW: *χ*^2^ = 0.25; *p*=0.97; Figure 2(f)) in the hippocampus of newborn offspring did not differ statistically between the groups. However, the DOPAC concentration differed between the groups according to the KW test (KW: *χ*^2^ = 7.90; *p*=0.048), with the PS offspring demonstrating a higher concentration compared to the control offspring (*p*=0.04; Figure 2(e)). Post hoc analysis indicated no statistical differences between the groups for males (*p*=0.19) and females (*p*=0.49), separately.

The concentration of norepinephrine in the hippocampus of newborn rats did not differ significantly between the groups (KW: *χ*^2^ = 3.57; *p*=0.31; Figure 2(g)).

### 3.3. BDNF and Neuropeptides Analysis in the Whole Brain

**Figure 3:**
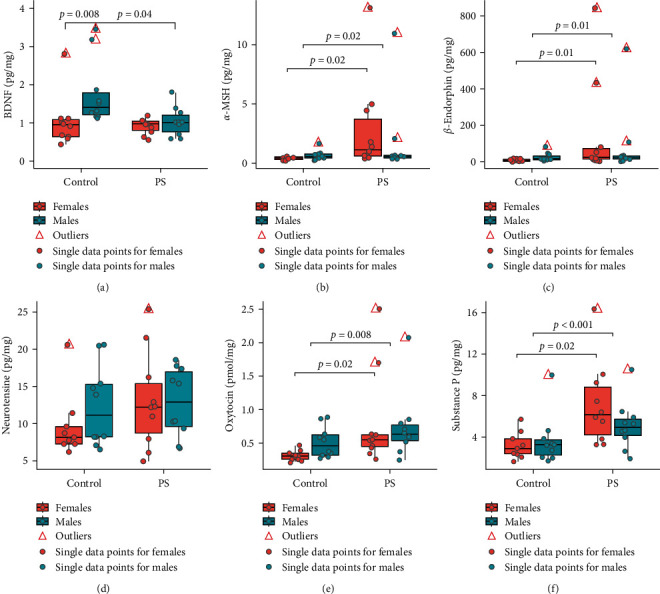
Concentration of BDNF and some neuropeptides in the newborn rat's whole brain altered after exposure to ultrasound prenatal stress (*n* = 40 : 10 control males, 10 control females, 10 PS males, 10 PS females): (a) BDNF concentration in the whole brain; (b) *α*-MSH concentration in the whole brain; (c) *β*-endorphin concentration in the whole brain; (d) neurotensine concentration in the whole brain; (e) oxytocin concentration in the whole brain; (f) substance P concentration in the whole brain. Data are expressed as med (Q1; Q3) in boxplots; red triangles—outliers; colored dots—single data points (red—females, blue—males); red boxes—data on females; blue boxes—data on males; control, control group; PS, prenatal stress group; *α*-MSH, *α*-Melanocyte-stimulating hormone.

The KW test indicated significant differences between the groups by the total BDNF concentration in the whole brain (KW: *χ*^2^ = 16.39; *p* < 0.001; Figure 3(a)). We uncovered differences between the sexes in the control group. The control males demonstrated an increased concentration of BDNF in the brain tissue compared to the control females (*p*=0.008). In addition, PS significantly decreased the BDNF concentration in males (*p*=0.04) but not in females (*p*=0.99), which led to the elimination of the sex differences of the BDNF concentration in PS offspring (*p*=0.93).

The KW test also revealed differences between the groups in *α*-MSH concentration (KW: *χ*^2^ = 10.19; *p*=0.01). US PS increased the level of *α*-MSH in the offspring (*p*=0.02); post hoc analysis indicated that *α*-MSH concentration increased only in females (*p*=0.02): males (*p*=1.00) (Figure 3(b)).

The KW test demonstrated significant differences between the groups by *β*-endorphin concentration (KW: *χ*^2^ = 11.37; *p*=0.01). US PS increased *β*-endorphin levels in the offspring (*p*=0.01): post hoc analysis revealed that *β*-endorphin concentration increased only in females (*p*=0.01): males (*p*=0.95) (Figure 3(c)).

US PS produced no effect on the neurotensin concentration in the brain of the newborn offspring (KW: *χ*^2^ = 2.49; *p*=0.48) (Figure 3(d)).

The KW test revealed statistical differences between the groups in oxytocin concentration (KW: *χ*^2^ = 12.06; *p*=0.007). US PS increased oxytocin concentration in the offspring (*p*=0.008); post hoc analysis indicated that oxytocin concentration increased only in females (*p*=0.02): males (*p*=0.67) (Figure 3(e)).

The KW test also showed significant differences between the groups by substance P concentration (KW: *χ*^2^ = 12.22; *p*=0.007). US PS increased substance P concentration in the offspring (*p*  < 0.001); post hoc analysis revealed that substance P concentration increased only in females (*p*=0.02): males (*p*=0.23) (Figure 3(f)).

### 3.4. Correlation Analysis

The results of correlation analysis between the studied biochemical parameters of the brain demonstrated the presence of multiple correlations. Reliable results of the analysis are presented in Table 1.

Correlation analysis showed a direct relationship between serotonin, dopamine, and their metabolites in the frontal cortex and hippocampus in the control and PS offspring, which was expected and confirmed the relevance of the analysis.

**Figure 4:**
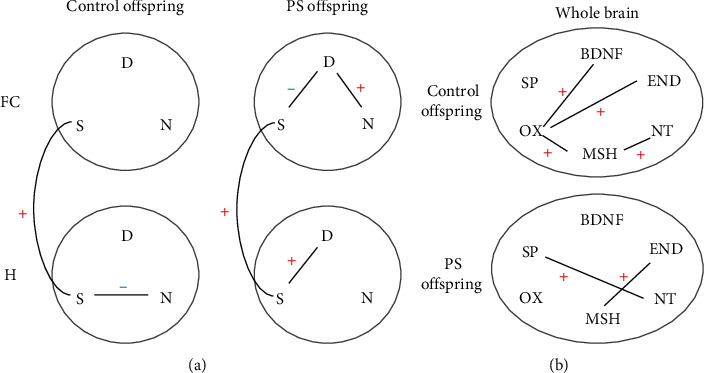
Biochemical correlation networks in the frontal cortex and hippocampus (a) and in the whole brain (b). A black line is displayed when Spearman's correlation coefficient was *r* > 0.5 or *r* <−0.5, and statistical significance *p*  < 0.01 between biochemical systems; red plus: positive correlation; blue minus: negative correlation; PS, prenatal stress; H, hippocampus; FC, frontal cortex; S, serotonergic system; D, dopaminergic system; N, noradrenergic system; BDNF, brain-derived neurotrophic factor; MSH, *α*-melanocyte-stimulating hormone; SP, substance P; END, *β*-endorphin; NT, neurotensin; OX, oxytocin.

In our study, we were interested in identifying dynamic networks among neurotransmitters, so, we constructed correlation networks in which we presented the relationships between different neurotransmitter systems (Figure 4(a)). Multiple correlations were found between indicators of dopaminergic and serotonergic system activity, which were positive in the hippocampus and negative in the frontal cortex. A positive relationship between the dopaminergic and noradrenergic systems was shown in the PS offspring, and a negative correlation was found between the noradrenergic and serotonergic systems in the controls. Another important result is the detection of multiple correlations between the indicators of serotonin metabolism activity of the frontal cortex and the hippocampus, which are positive and expressed to a greater extent in the PS offspring. Summarizing the data obtained, it should be noted that after PS exposure, the correlation network of neurotransmitters became more complex in the frontal cortex and that changes in the structure of the hippocampal network were also detected.

A change in the pattern of interactions between brain neuropeptidergic systems after PS exposure was shown (Figure 4(b)). Positive interactions between brain oxytocin and other neuropeptides or BDNF were detected in the control offspring. But they had disappeared in the PS offspring. In addition, correlations between neuropeptides appeared in females after PS exposure, which was not the case in the control females.

### 3.5. PCA

**Figure 5:**
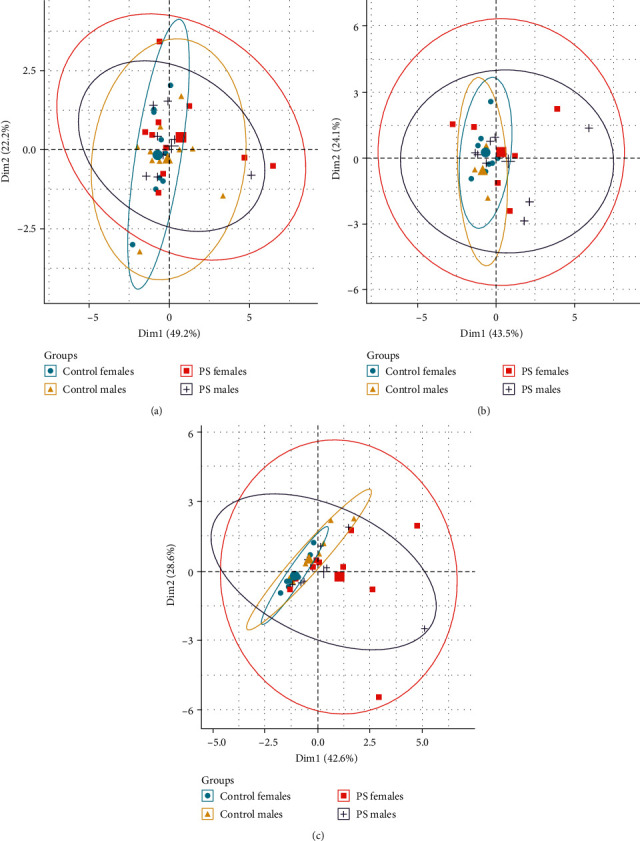
PCA of all biochemical parameters stratified according to group and sex: (a) score plot for the frontal cortex; (b) score plot for the hippocampus; (c) score plot for the whole brain; PS, prenatal stress; Dim1, component 1; Dim2, component 2.

Group and sex were considered qualitative variables, whereas the biochemical variables tested were considered quantitative variables in the PCA analysis. The analysis showed no difference between groups and sexes in the frontal cortex, hippocampus, and whole brain (Figure 5).

**Figure 6:**
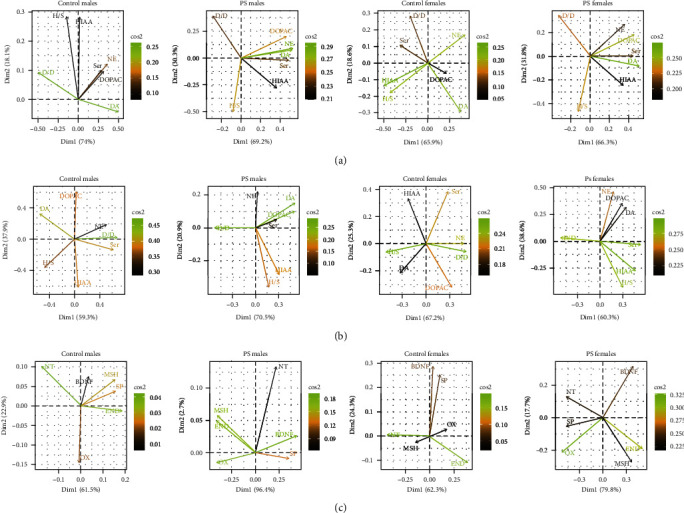
PCA of all biochemical parameters stratified according to sex and group: (a) biplots of the variables for the frontal cortex; (b) biplots of the variables for the hippocampus; (c) biplots of the variables for the whole brain; PS: prenatal stress; Dim1: component 1; Dim2: component 2; cos2: the degree of the variable representation in each component—high cos2 attributes are colored in green, low cos2 attributes have a black color; DA, dopamin; DOPAC, 3,4-dihydroxyphenylacetic acid; D/D, DOPAC/dopamine ratio; Ser, serotonin; HIAA, 5-hydroxyindoleacetic acid; H/S, HIAA/serotonin ratios; NE, norepinephrine; BDNF, brain-derived neurotrophic factor; MSH, *α*-melanocyte-stimulating hormone; SP, substance P; END, *β*-endorphin; NT, neurotensin; OX, oxytocin.

After stratification according to group and sex, PCA revealed that the first (Comp 1) and second (Comp 2) principal components explained most of the variance. In the frontal cortex, Comp 1 and Comp 2 explained 74% and 18.1% of the variance in the control males, 69.2% and 30.3% of the variance in the PS males, 65.9% and 18.6% of the variance in the control females, and 66.3% and 31.8% of the variance in the PS females (Figure 6(a)). PS males and females were similar in the loading patterns of Comp 1 and Comp 2. In this case, Comp 1 included dopamine, serotonin, and their metabolites DOPAC and HIAA, which had a positive relationship with Comp 1. The Comp 2 included the DOPAC/dopamine and HIAA/serotonin ratios. The control males and females had different loadings on Comp 1 and Comp 2. In the control males, Comp 1 included indicators of dopamine metabolism, and Comp 2 was characterized mainly by indicators of serotonin metabolism. In the control females, a mixed load on Comp 1 and Comp 2 was observed.

In the hippocampus, Comp 1 and Comp 2 explained 59.3% and 37.9% of the variance in the control males, 70.5% and 20.9% of the variance in the PS males, 67.2% and 25.3% of the variance in the control females, and 60.3% and 38.6% of the variance in the PS females (Figure 6(b)). For the control males and females, a high load of norepinephrine, DOPAC/dopamine ratio, and HIAA/serotonin ratio on Comp 1 was shown. Comp 2 included the metabolites HIAA and DOPAC, which had a different relationship with Comp 2 in the control males and females. The components had a different load in the PS offspring: Comp 1 was characterized by the serotonin and DOPAC/dopamine ratio; Comp 2 included the norepinephrine and HIAA/serotonin ratio. It should also be noted that PS males and females were more similar to each other with respect to the Comp 1 and Comp 2 loads than the control males and females.

In the whole brain, Comp 1 and Comp 2 explained 61.5% and 22.9% of the variance in the control males, 96.4% and 2.7% of the variance in the PS males, 62.3% and 24.3% of the variance in the control females, and 79.8% and 17.7% of the variance in the PS females (Figure 6(c)). Comp 1 and Comp 2 had different neuropeptide and BDNF loadings in all groups. PS females and males did not have similar loading on components, which has been shown for neurotransmitter systems.

## 4. Discussion

Our study demonstrated for the first time that US PS alters the concentration of some neurotransmitter system metabolites, neuropeptides, and neurotrophin in the brain of newborn rats, suggesting their involvement in the mechanism of action of US PS on neurodevelopment.

First of all, it was shown that US PS affects the neurotransmitter systems of the hippocampus but not the frontal cortex, indicating a greater vulnerability of the hippocampus to this exposure. US PS increased the concentration of serotonin in the hippocampus of newborn rats, but it did not significantly alter the concentration of its metabolite HIAA or its ratio, indicating the absence of changes in serotonin metabolism. Other studies have also previously observed alterations in the activity of the serotonergic system in some brain regions of newborn and juvenile animals under the influence of PS, but the results of those studies have been contradictory. Some studies have shown an increase in serotonin metabolism in the hippocampus of 21 PND mice after the restrainer PS [46], in the hippocampus of rats (35 PND) under the influence of PS induced by cramped housing conditions and intramuscular injections of saline [47], in the cortex of rats (16 PND) and in the hypothalamus of rats (23 PND) after the PS induced by daily subcutaneous saline injections [48], in the preoptic region of female rats (10 PND), and in the mediobasal hypothalamus of same-age males after the restrainer PS [49]. At the same time, other studies have shown a decrease in serotonin metabolism in the ventral hippocampus of rats at 4 weeks of age in a dexamethasone PS model [50], in the neocortex, hippocampus, hypothalamus, and midbrain of rats at 3 weeks of age in the dexamethasone model of PS [51], and in the hypothalamus of infant rats (9 PND) after PS induced by daily subcutaneous saline injections [48]. Some studies have described the effect of PS on the serotonin metabolism in fetuses during late pregnancy. In one study, PS induced by cramped housing conditions and daily intramuscular injections of saline in pregnant female rats resulted in increased total brain serotonin level at 20 gestational days (GD) and HIAA level at 20 and 21 GD, indicating the amplification of serotonin metabolism [52]. Another study revealed that chronic unpredictable PS increases hippocampal and hypothalamic serotonin levels, decreases hippocampal HIAA, and decreases hippocampal and hypothalamic HIAA/serotonin ratios in fetal rats at 20 GD, indicating a decrease in serotonin metabolism [53].

In our study, we also demonstrated an increase in DOPAC concentration in the hippocampus of neonatal rats, indicating a possible increase in dopamine metabolism. However, we did not uncover any DOPAC/dopamine ratio changes. A number of animal studies have shown the effect of PS on the dopaminergic system in offspring during early age. Some studies have demonstrated a decrease in the dopamine metabolism level in the hippocampus of rats (21 PND) after the restrainer PS [54], in the preoptic region of male and female rats (10 PND) after the restrainer PS [55], and also a decrease in the dopamine metabolism, accompanied by lower DOPAC levels, in the hypothalamus of rats (21 PND) in a dexamethasone PS model [51]. In addition, dopamine levels decreased in the preoptic region of the female rats (10 PND) after PS [49]. However, some data conversely indicated an increase in dopamine metabolism in the rat striatum (21 PND) in a dexamethasone PS model [51]. Additionally, it was shown that the restrainer PS stimulates the production of extracellular dopamine in the nucleus accumbens of rats at 30–35 PND [56] and in the mediobasal hypothalamus of female rats at 10 PND [49]. Therefore, our study centered on the possible effect of PS on neurotransmitter systems and confirmed the data of some previous studies. However, the peculiarities of this effect were variable. The consequences of PS exposure probably depend on additional factors such as sex, age, and the species of animals, as well as on the type of applied PS [19, 48, 49].

Our study also revealed that US PS leads to an increase in some neuropeptides' concentration in the brain of newborn rats. Significant results were obtained for *α*-MSH, *β*-endorphin, oxytocin, and substance P. Nevertheless, statistically significant changes in concentration were noted only in females. A limited number of studies have focused on research into neuropeptide concentrations in the brains of newborn offspring after PS. However, it has been shown that some neuropeptide systems may change after PS at an early age.

For example, a study of the *β*-endorphin level in the hypothalamus of rats at the age of PND 10 demonstrated an increase of *β*-endorphin in those animals that were exposed to PS during 2–6 GD and during 7–11 GD. However, it was also shown that the longer PS of 2–16 GD did not affect the *β*-endorphin concentration in the hypothalamus [57]. The same authors uncovered a decrease in the mu-opioid receptor density in different brain regions of the rats' offspring (PND 10) after exposure to PS at 2–6 GD, 12–16 GD, and 2–16 GD [58]. One study noted decreased *β*-endorphin levels in the hypothalamus of male fetuses after immobilization stress at 20 GD. Opioid levels in the pituitary gland were initially increased in the fetuses of both sexes, but they later decreased only in male fetuses [59]. Thus, data from these studies, together with our results, suggest that endogenous opioids may contribute to the etiology of the impairments that originate after PS.

For the first time, we have revealed changes in the oxytocinergic system of the brain of newborn rats after PS. Quite a few researchers have studied this aspect in adult animals. For example, it has been shown that PS can lead to a decrease in the number of OT-positive magnocellular neurons and simultaneously induce an increase in anxiety and aggressiveness in adult rats [60]. In addition, it was demonstrated that PS decreases the oxytocin receptor expression in the cerebral cortex of adult male mice [61], decreases oxytocin expression in the paraventricular nucleus, and increases the binding to the oxytocin receptor in the central amygdala in male rats [62]. It has also been shown that adult voles whose mothers have been subjected to immobilization stress experience a decrease in the number of oxytocin immunoreactive neurons in the paraventricular and supraoptic nuclei of their hypothalamus [63].

The effect of PS on *α*-MSH and substance P level is also poorly studied. It is known that *α*-MSH plays an important role in fetal development. It forms in the fetal rat brain and pituitary gland as early as on the 16th day of pregnancy. In the rat pituitary gland, the *α*-MSH level increases after birth; it has been suggested that *α*-MSH plays the role of major pituitary peptide throughout the postnatal life [64, 65]. Therefore, changes in its level in the brain after US exposure in the prenatal period could potentially influence CNS development and, ultimately, the formation of the behavioral phenotype. Some evidence suggests that substance P may be involved in the stress response, because, in rats subjected to stress, release of the substance P in the medial amygdala was demonstrated [66]. Nevertheless, it remains unknown how substance P may be involved in the response to PS.

It is reasonable to assume that these neuropeptides play a role in neurodevelopment and mediate the effect of US PS on neurodevelopment, since the changes in the neuropeptide systems are observed immediately after birth. Particularly, neuropeptides can modulate neurodevelopment because they regulate synapse formation, neuronal proliferation, and differentiation during the early stage of brain development [22]. Because of that, the proposition that they play a role in the formation of the behavioral phenotype of animals following the influence of external factors is plausible.

We have found evidence of increased serotonin and DOPAC concentrations only in the hippocampus and under the influence of PS. At the same time, the concentration of neurotransmitters and their metabolites remained unchanged in the frontal cortex. However, the network of neurotransmitter systems had clearly added in complexity in the frontal cortex, indicating some changes in this structure under the influence of PS. In addition, the pattern of connections had changed in the networks of hippocampal neurotransmitters and neuropeptides in the whole brain. These results suggest a change in the balance between the biochemical systems of the brain during prenatal development. Interestingly, our study observed activation in both the neurotransmitter systems and in the neuropeptidergic systems. Presumably, this unidirectional change may indicate impaired feedback of the mechanisms that regulate the activity of different neurochemical systems, including the links that are common to systems of different ergicities. Further research is needed to identify them.

The important observation of our study was the detection of the decrease in the BDNF concentration in the brain of newborn males under the influence of US PS. A similar pattern has been observed in other numerous animal studies using different PS paradigms. In these studies, BDNF gene transcriptional and epigenetic alterations characterized by the decrease in the BDNF gene expression and the increase in its methylation in the brain tissue of young and adult rats and mice were indicated [67–74]. Studies that have examined alterations of the total brain BDNF concentration support the data resulting from expression studies [19, 73]. However, the results of such studies depended on the type of PS, the sex of the fetus, the line of the animal, and the period of pregnancy when the females experienced stress [15, 19, 73, 75]. The decrease in th BDNF biosynthesis level in the brain was observed not only in adults but also in newborns [76]. For example, in PS rats, BDNF was reduced in the olfactory bulbs and hippocampus by 1–5 PND [77]. BDNF expression was also significantly reduced in the prefrontal cortex and hippocampus of newborn rats (7 PND) exposed to PS [17]. Certain areas of the brain, such as the hippocampus and the prefrontal cortex, are the most vulnerable areas to PS exposure [15]. However, the decrease in BDNF concentration is also characteristic of the total brain [19, 75]. Since the stress-related neuropathology is not limited to that one particular area of the brain [19], in our study, we focused on research into total brain tissue and uncovered changes in the BDNF and neuropeptides concentrations not in the specific brain region, but in the whole brain.

The important factor that affects BDNF concentration in the brain is gender, since some BDNF functions or mechanisms of action differ by gender [78]. It has been suggested that sex hormones can modulate BDNF activity [15, 78]. It has been demonstrated experimentally that gonadal steroids can affect BDNF expression in the brain, as neonatal administration of exogenous estradiol induces the level of changes in BDNF expression [79]. Because of its sex-specific expression, it has been suggested that BDNF mediates different functions in males and females. One study provides support for this hypothesis. In particular, it was demonstrated in it that BDNF knockout in the forebrain of mice induces hyperactive behavior only in male mice. In contrast, BDNF knockout female mice showed anxious and depressive-like behavior, which was not characteristic of the males [80]. In addition, different kinds of stress exposure can alter BDNF expression in the brains of males and females in different ways [78], indicating the difference in the stress response between the sexes. Our study found that male rats (1 PND) demonstrate higher total BDNF concentrations than females. The results of some previous studies are consistent with this pattern. For example, newborn male rats demonstrated higher BDNF gene expression compared to females in some regions of the hippocampus: in the dentate gyrus, the C1, and C3 areas [79, 81]. Other data shown that female rats had a higher BDNF level in the hippocampus, ventromedial hypothalamus, and cortex compared to the males [82–85]. One study shows a higher concentration of BDNF in the dorsal dentate gyrus of the hippocampus in male rats compared to females [86]. However, only a limited number of studies are devoted to uncovering the sex differences in the BDNF level in the brains of newborn animals, so, this aspect needs study in further detail.

Biochemical profiles appear to be specific to each brain region, but the control and PS rats appear to have different trajectories, which are also sex-dependent. In our study, the PCA analysis showed that PS offspring had fewer differences between the sexes in terms of the activity of the neurotransmitter systems of the frontal cortex and hippocampus compared with the control animals. This is an important observation in view of the data on the effects of US PS on behavior. Earlier studies found an altered behavioral phenotype in animals with the US PS experience; in particular, feminization-of-play behavior was observed in juvenile rat males [28]. The present study has demonstrated that this phenomenon exists at the level of neurotransmitter systems.

In our previous studies, we demonstrated that US PS leads to the development of psychopathological behavioral features in the offspring of rats in adulthood and early life, in particular to impaired social behavior and increased levels of anxiety. We have hypothesized the possible presence of autism-like behavioral traits in such animals [27, 28]. The new results in this study seem to indicate that US PS probably alters neurodevelopment, which correlates with the data on the pathophysiology of autism. Autism spectrum disorder is known to be a condition associated with impaired nervous system development [87, 88].

Therefore, it seems fair to assume that the identified biochemical alterations are rather pathological, but this point should be further investigated in the context of the influence of the environment in the postnatal period, since, according to some authors [89], PS can create the conditions for increased susceptibility to external environmental influences in the postnatal period, while this susceptibility can lead to negative or positive effects, depending on the environmental character. Learning is an important factor involved in the developmental process here. The brain is highly plasticity and adapts quickly to new experiences, and in the course of during development, it can undergo both progressive developmental stages and regressive ones [90]. The training process can have different outcomes, depending on age, the specific function being trained, and the brain structures in which changes occur [90]. The developing brain has a greater capacity for plasticity compared to the adult brain [91]. As a result, the features found in rats with UW PS experience need to be investigated in the context of the influence of the postnatal environment, and this includes both negative environmental factors (e.g., early life stress) and positive ones (e.g., enriched environment, learning). However, this issue is the subject of future research.

Despite the importance of the obtained results, our study has some limitations. Caution should be exercised as it relates to the data for dopamine and DOPAC, because the obtained data are at the lower threshold of the method's resolving power. Another limitation is the fact that we determined the concentration of the neuropeptides and BDNF in the whole brain. Although these results are also important, in future experiments, it would be necessary to measure the concentration of the studied parameters in the separate brain structures. In addition, the age dynamics of the concentrations should be further studied, since biochemical parameters may change during maturation. It is particularly important to determine these indicators in adult animals, as it is relevant to study the relationship between biochemical scores and behavioral parameters. The obtained data may be of practical importance, since it could reboost our capacity to model certain mental disorders based on US PS exposure.

## 5. Conclusions

For the first time in practice, our study has demonstrated that US PS increases the levels of serotonin and the dopamine metabolite DOPAC in the hippocampus, decreases the level of the neurotrophin BDNF, and increases the levels of the neuropeptides *α*-MSH, *β*-endorphin, oxytocin, and substance P in the whole brain of newborn rats. In addition, the pattern of interaction between different biochemical systems of the brain under the influence of US PS has been observed to alter. Since these systems play an important role in the process of neurodevelopment, as well as in the behavioral function, it seems legitimate to suggest their possible participation in the mechanism of behavioral phenotype alterations in animals under the influence of US PS. The results of our study provide substantial information on the possible neurochemical mechanisms of the PS action in the process of neurodevelopment. In addition, these data may be useful in determining how to develop a psychopathology model based on US PS.

## Figures and Tables

**Table 1:** Correlation analysis of the relationship between the brain biochemical indicators.

Group	Biochemical indicator 1	Biochemical indicator 2	*R*	*p* Value
Brain regions—hippocampus and frontal cortex (neurotransmitter systems)				

Control offspring	Dopamine (FC)	DOPAC (FC)	0.61	0.005
	DOPAC/dopamine ratio (FC)	Dopamine (FC)	−0.57	0.0096
	HIAA/serotonin ratio (FC)	HIAA (FC)	0.86	<0.001
	DOPAC/dopamine ratio (H)	Dopamine (H)	−0.82	0.002
	HIAA/serotonin ratio (H)	Norepinephrine (H)	−0.58	0.008
	HIAA/serotonin ratio (H)	HIAA (H)	0.59	0.007
	HIAA/serotonin ratio (H)	Serotonin (H)	−0.64	0.003

PS offspring	DOPAC (FC)	Norepinephrine (FC)	0.60	0.008
	Dopamine (FC)	DOPAC (FC)	0.70	0.001
	Serotonin (FC)	HIAA (FC)	0.81	<0.001
	Dopamine (H)	HIAA/serotonin ratio (FC)	0.67	0.007
	Dopamine (H)	DOPAC (H)	0.81	<0.001
	DOPAC/dopamine ratio (H)	HIAA (FC)	−0.66	0.009
	DOPAC/dopamine ratio (H)	Dopamine (H)	−0.82	<0.001
	HIAA (H)	HIAA/serotonin ratio (FC)	0.90	<0.001
	HIAA (H)	DOPAC (H)	0.70	0.005
	HIAA (H)	Dopamine (H)	0.80	<0.001
	Serotonin (H)	Dopamine (H)	0.72	0.003
	Serotonin (H)	HIAA (H)	0.68	0.001
	HIAA/serotonin ratio (H)	HIAA/serotonin ratio (FC)	0.67	0.002
	HIAA/serotonin ratio (H)	HIAA (H)	0.63	0.004

Control males	HIAA/serotonin ratio (FC)	HIAA (FC)	0.85	0.003

Control females	HIAA/serotonin ratio (FC)	HIAA (FC)	0.91	<0.001
	HIAA (H)	HIAA (FC)	0.82	0.007

PS males	HIAA/serotonin ratio (FC)	DOPAC (FC)	−0.83	0.008
	Serotonin (FC)	HIAA (FC)	0.83	0.006
	Dopamine (H)	DOPAC (H)	0.92	0.001
	HIAA (H)	HIAA/serotonin ratio (FC)	0.90	<0.001
	HIAA (H)	DOPAC (H)	0.83	0.008

PS females	HIAA (H)	HIAA/serotonin ratio (FC)	0.90	<0.001

Whole brain (BDNF and neuropeptides)				

Control offspring	Oxytocin	BDNF	0.72	<0.001
	Oxytocin	*α*-MSH	0.62	0.005
	Oxytocin	*β*-Endorphin	0.71	<0.001
	Neurotensin	*α*-MSH	0.80	<0.001

PS offspring	*β*-Endorphin	*α*-MSH	0.70	<0.001
	Substance P	Neurotensin	0.82	<0.001

Control males	Neurotensin	*α*-MSH	0.81	0.008

PS males	Substance P	Neurotensin	0.83	0.006

PS females	*β*-Endorphin	*α*-MSH	0.82	0.007
	Substance P	Neurotensin	0.85	0.003

*α*-MSH, *α*-melanocyte-stimulating hormone; BDNF, brain-derived neurotrophic factor; DOPAC, 3,4-dihydroxyphenylacetic acid; H, hippocampus; HIAA, 5-hydroxyindoleacetic acid; PS, prenatal stress; FC, frontal cortex.

## Data Availability

The data that support the findings of this study are available from the corresponding author upon reasonable request.
